# Foliar Application of Polyamines Modulates Winter Oilseed Rape Responses to Increasing Cold

**DOI:** 10.3390/plants9020179

**Published:** 2020-02-01

**Authors:** Elžbieta Jankovska-Bortkevič, Virgilija Gavelienė, Vaidevutis Šveikauskas, Rima Mockevičiūtė, Jurga Jankauskienė, Dessislava Todorova, Iskren Sergiev, Sigita Jurkonienė

**Affiliations:** 1 Nature Research Centre, Laboratory of Plant Physiology, Akademijos Str. 2, LT-08412 Vilnius, Lithuania; virgilija.gaveliene@gamtc.lt (V.G.); vaidevutis.sveikauskas@gamtc.lt (V.Š.); rima.mockeviciute@gamtc.lt (R.M.); jurga.jankauskiene@gamtc.lt (J.J.); sigita.jurkoniene@gamtc.lt (S.J.); 2 Bulgarian Academy of Sciences, Institute of Plant Physiology and Genetics, Acad. G. Bonchev Str. Bl. 21, Sofia BG-1113, Bulgaria; dessita@bio21.bas.bg (D.T.); iskren@bio21.bas.bg (I.S.)

**Keywords:** acclimation, *Brassica napus*, canola, cold stress, cold tolerance, crop plants, putrescine, spermidine, spermine

## Abstract

Cold stress is one of the most common abiotic stresses experienced by plants and is caused by low temperature extremes and variations. Polyamines (PAs) have been reported to contribute in abiotic stress defense processes in plants. The present study investigates the survival and responses of PA-treated non-acclimated (N) and acclimated (A) winter oilseed rape to increasing cold conditions. The study was conducted under controlled conditions. Seedlings were foliarly sprayed with spermidine (Spd), spermine (Spm), and putrescine (Put) solutions (1 mM) and exposed to four days of cold acclimation (4 °C) and two days of increasing cold (from −1 to −3 °C). Two cultivars with different cold tolerance were used in this study. The recorded traits included the percentage of survival, H^+^-ATPase activity, proline accumulation, and ethylene emission. Exogenous PA application improved cold resistance, maintained the activity of plasma membrane H^+^-ATPase, increased content of free proline, and delayed stimulation of ethylene emission under increasing cold. The results of the current study on winter oilseed rape revealed that foliar application of PAs may activate a defensive response (act as elicitor to trigger physiological processes), which may compensate the negative impact of cold stress. Thus, cold tolerance of winter oilseed rape can be enhanced by PA treatment.

## 1. Introduction

The growth and development of plants is strongly dependent on the surrounding environment. Exposure to non-optimal temperature, humidity, salinity, and acidity causes stress responses in plants. A series of morphological, physiological, biochemical, and molecular changes can be triggered in plants under the effect of abiotic stresses and negatively affect plant growth and productivity of crops [1,2,3,4].

One of the most common abiotic stresses experienced by plants is cold stress caused by low temperature extremes and variations. Abrupt cold events and daily thermal fluctuations frequently occur in the temperate biomes in autumn–winter periods. Overwintering plants have developed the ability to acclimate to cold via exposure to low non-freezing temperatures. During the process of cold acclimation, plants undergo physiological changes that promote plant tolerance to cold and increase survival [5,6,7]. However, the genetic characteristics of the species do not always determine the metabolism rearrangement and the tolerance to sudden temperature fluctuations in autumn, winter, and spring. The natural process of acclimation is not capable of ascertaining good wintering of winter oilseed rape (*Brassica napus* L. ssp. *Oleifera biennis* Metzg) cultivated in the countries of the temperate zone, especially of cultivars introduced from warmer countries to temperate regions such as the Baltic States [8,9,10]. Oilseed rape is a commercially important crop known to have an economic relevance for food oil, biofuel, green manure, feed for livestock, and other market needs. The minimization of the loss of yield is one of the main objectives for plant and crop specialists, and it is immediate because of the growing demands for food and feed. Winter hardiness and freezing tolerance are a major concern for improving production consistency in many regions of the winter oilseed rape growing countries [11]. The research on the mechanism of plant abiotic stress and its control is one of the most important scientific fields to date [12,13,14].

The amino acid proline is thought to be one of the biomarkers in plant response to cold [15,16]. Proline plays several major roles during cold stress. It protects cells from damage by acting as an osmolyte–cryoprotectant, signaling molecule, and stabilizer of subcellular structures [17,18,19]. High levels of proline stored under acclimation and stress conditions are beneficial during stress recovery and provide a supply of energy to resume growth once the stress is relieved [5,18]. Other physiological traits involved in the response of plants to increasing cold stress could be the activity of the plant cell membrane H^+^-ATPase. The changes in ATPase activity following cold acclimation and cold stress have been observed by authors of [16,20,21,22,23]. The H^+^-ATPase is very sensitive to cold stress and closely related to cell membrane defense from injury. The levels of the plant hormone ethylene can be considered as one of traits of low temperature transient changes. It is known that production of ethylene decreases or stops with injury and death of cells [24,25,26,27]. On the contrary, greater ethylene biosynthesis has been shown in low temperature treated winter rye, tobacco, tomato, and oilseed rape [2,10,28]. Thus, ATPase activity, proline, and ethylene levels in plant tissues are the main traits of the plant response to cold stress leading to plant survival in harsh winter conditions.

There are several ways to increase the stress resistance of plants, for instance genetic modification and the exogenous use of physiologically active substances. Scientists have been trying to overcome the impacts of environmental stresses, including cold, by employing different strategies, such as foliar application of plant growth regulators, osmoprotectants, and organic and inorganic nutrients, which are efficient, economical, and environmentally sound approaches [29,30]. There is evidence in the literature of the beneficial effects of synthetic growth regulators (phytohormones, amino acids, polyamines (PAs), fungicides, retardants, and others) on plant stress reduction [13,31,32]. PAs, generally putrescine (Put), spermidine (Spd), and spermine (Spm), are polycationic low molecular weight compounds present in living organisms. PAs are known to be involved in plant development, cell division, embryogenesis, fruit development, dormancy termination, regulation of aging, and temperature stress processes [1,33,34]. Many authors indicate that PA accumulation is an important component of a response to cold stress in different crop plant species and that PAs play a significant role in counteracting stress [33,35,36,37]. Studies of transgenic plants showed that the overproduction of PAs coincided with better stress tolerance [38]. Recent studies using exogenous PAs have shown that PAs enhanced wheat and maize tolerance to drought by increasing endogenous PAs [34,39]. PAs are closely associated with plant growth, stability of nucleic acids and membrane structure, stress resistance, and even plant survival [40,41,42]. The application of exogenous Spm maintained high levels of endogenous Spm and Spd, inhibiting Put accumulation and reducing chilling damage [43,44]. Another opinion is that Put may accumulate as a defense response of plants to chilling damage, because Put accumulation has been found to be positively correlated with the cold resistance of plants [45]. Therefore, it is appropriate to investigate the specificity of individual PAs in the response of plants to cold.

There is some evidence that PAs can inhibit ethylene biosynthesis in plants, and the relationship between PAs and ethylene seems to be dependent on the plant species [37,46,47]. Under stress conditions, ethylene causes growth inhibition, regulates defense processes, mostly in full-grown leaves, and growth in young leaves. To counteract cold stress, plants synthesize osmolytes and perform osmotic adjustment in cold tolerance [48]. Some reports have indicated that exogenous PAs induced proline accumulation [41,49]. Proline is important compatible osmolyte that maintains turgor, stabilizes cell molecular structure, and acts as a source of energy and nitrogen under various stress conditions [41]. It is still unknown how the accumulation of cryoprotectors such as proline is impacted by PAs in plant tissues and, thus, influences frost resistance. Different studies indicate that the cell membrane systems of the cell are the primary site of freezing injury in plants and it reflects on cell permeability [50,51,52]. Freezing injury causes the altered physical and chemical composition of cell membranes and the activity of transport enzymes [53]. The activities of the cell membrane ATPases are dependent upon the status of the membranes as influenced by temperature [54]. The physical and chemical properties of membranes can be modified by PAs, which are polycationic molecules and can bind to the phospholipid site of the cell membrane to change their stability and improve cold resistance [55]. Such membrane fluidity modification could affect the activity of H^+^-ATPase located at the plasma membrane. However, it is still not fully understood how PAs regulate stress responses. To find out how PAs affect plant response to low temperature for increasing plant plasticity, it is crucial to investigate the physiological traits plants employ for tolerating cold stress. Different plant species respond differently to cold during hardening. In order to solve the problems of acclimation (preparation for wintering) and wintering under difficult (harsh) and varying meteorological conditions, it is appropriate to search for an effective tool to control these processes and adapt to temperate climate conditions.

We hypothesized that foliar application of PAs would change physiological traits of winter oilseed rape occurring after induced acclimation and increasing cold stress. A simulation model of harshening conditions of autumn–winter cold acclimation (4 °C) and increasing cold (from −1 to −3 °C), was used to assess undergoing physiological symptoms. The recorded traits included the percentage of survival, H^+^-ATPase activity, proline metabolism, and ethylene emission.

The aim of this study was to test the role of exogenously applied PAs (Spd, Spm, and Put) in mitigating adversities of increasing cold stress and to assess the effect on physiological traits of cold response in winter oilseed rape.

## 2. Results

### 2.1. Survival

**Figure 1:**
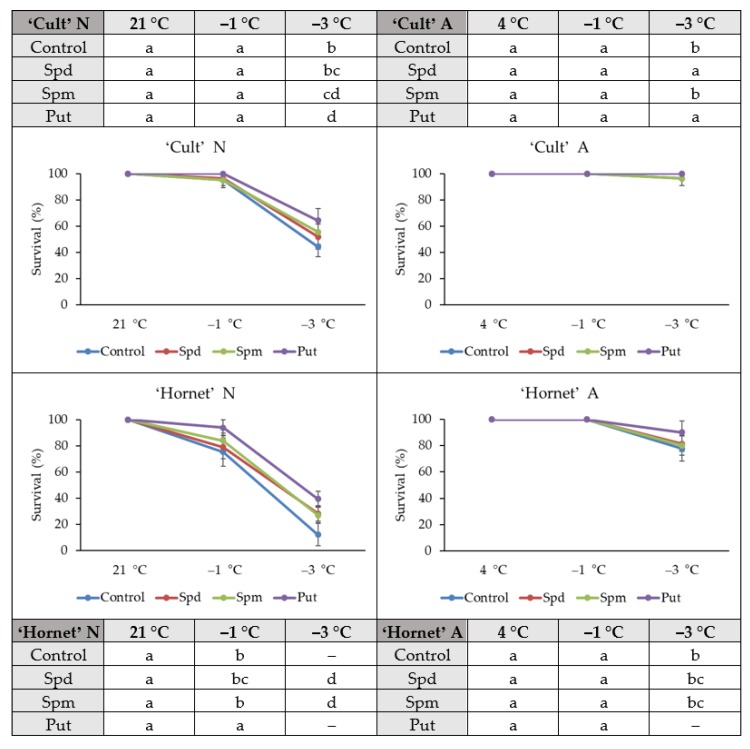
The effect of polyamine (PA) application, cold acclimation (4 °C, 4 days), and increasing cold treatment (from −1 to −3 °C, 2 days) on winter oilseed rape survival. Error bars represent the standard deviation of the mean. Control, Spd, Spm, and Put indicate the type of application (spermidine, spermine, and putrescine, respectively). Capital letters N and A indicate non-acclimation and acclimation, respectively. Different lowercase letters indicate statistically significant difference (*p* < 0.05).

The effect of each individual exogenous PA Spd, Spm, and Put (1 mM) on survival of non-acclimated (N) and acclimated (A) winter oilseed rape seedlings was assessed under simulated winter conditions (increasing cold from −1 to −3 °C, two days). Tested substances improved the resistance to increasing cold in N rape seedlings of both cultivars up to 27% (Figure 1). The strongest effect was characterized by Put. The number of surviving A cv. ‘Cult’ plants was significantly higher after Put and Spd treatment, when compared to control plants, and remained at one hundred percent. For A more sensitive to cold cv. ‘Hornet’ plants, significant differences were found in Put-treated plants, where Put application increased the survival up to 12%. Summing up, application of PAs increased the frost resistance both in N and A winter oilseed rape plants.

### 2.2. H^+^-ATPase Activity

No significant changes in H^+^-ATPase activity were found in N plants of both cultivars four days after foliar PA application. In the case of A plants, the H^+^-ATPase activity of the control plants was significantly lower than the activity of Spd-treated cv. ‘Cult’ (by 20%) and Put-treated cv. ‘Hornet’ (by 23%) plants.

**Figure 2:**
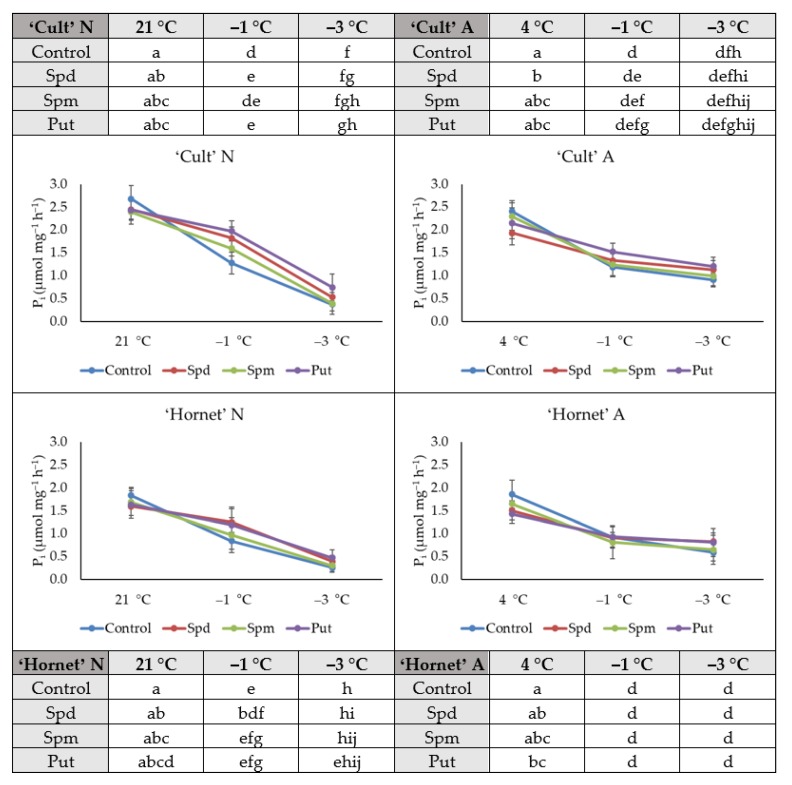
The effect of PA application, cold acclimation (4 °C, 4 days), and increasing cold treatment (from −1 to −3 °C, 2 days) on winter oilseed rape H^+^-ATPase activity. Error bars represent the standard deviation of the mean. Control, Spd, Spm, and Put indicate the type of application. Capital letters N and A indicate non-acclimation and acclimation, respectively. Different lowercase letters indicate statistically significant difference (*p* < 0.05).

After the first day of exposure to frost (−1 °C), H^+^-ATPase activity in N Spd- and Put-treated plants of cv. ‘Cult’ and Spd-treated cv. ‘Hornet’ plants was significantly higher when compared to control plants (Figure 2).

After the second day of increasing cold stress, the activity of this enzyme was significantly higher in Put-treated N cv. ‘Cult’ plants when compared to control plants.

Overall, the increasing cold treatment conditions (from −1 to −3 °C, two days) resulted in reduced H^+^-ATPase activity in all tested variants. H^+^-ATPase activity decreased by 85% in N control plants. In PA-treated N plants this change was smaller (69–84%). Acclimated control plants of both cultivars lost 62–68% of this enzyme activity under the effect of increasing cold. In plants affected by Spm, it decreased by 57–61% during increasing cold stress, and by 41–45% in plants affected by Spd and Put.

The obtained results showed that under increasing cold stress conditions the decrease of H^+^-ATPase activity both in N and A control plants was more intensive than that of PA-treated plants. Thus, the activity of H^+^-ATPase was more stable in plants treated with PAs under increasing cold conditions.

### 2.3. Proline Accumulation

**Figure 3:**
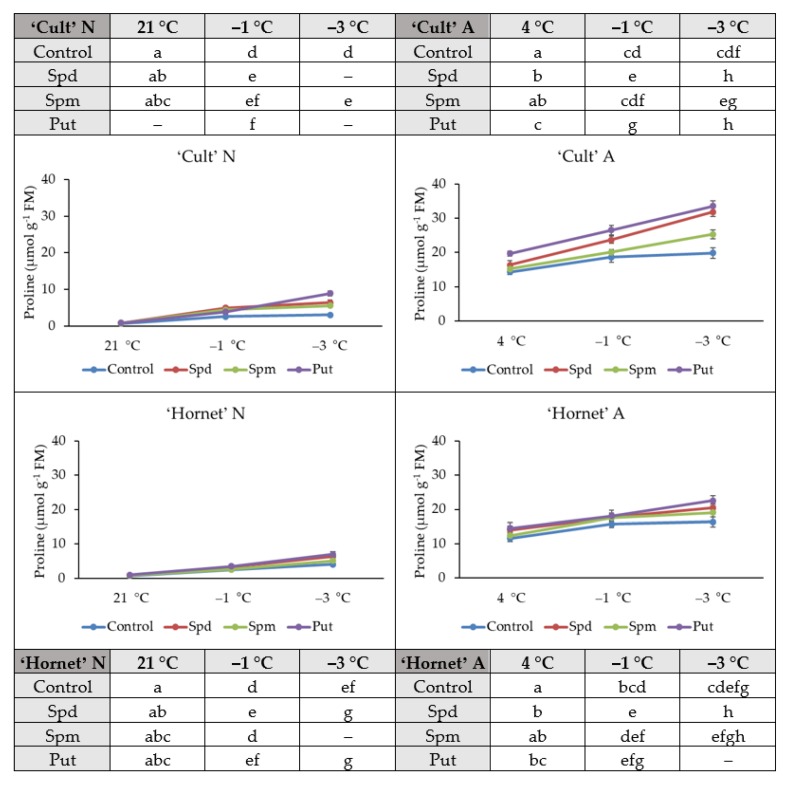
The effect of PA application, cold acclimation (4 °C, 4 days), and increasing cold treatment (from −1 to −3 °C, 2 days) on winter oilseed rape proline accumulation. Error bars represent the standard deviation of the mean. Control, Spd, Spm, and Put indicate the type of application. Capital letters N and A indicate non-acclimation and acclimation, respectively. Different lowercase letters indicate statistically significant difference (*p* < 0.05).

Our research data presented in Figure 3 showed how the proline content changed in the A and N winter oilseed rape leaves of the cvs ‘Cult’ and ‘Hornet’ treated with PAs and increasing cold stress.

PAs application affected the accumulation of proline both in A and N winter oilseed rape seedlings (Figure 3). The lowest proline concentration four days after PAs application was found in the N plants of both cultivars. Put treated N cv. ‘Cult’ plants accumulated significantly higher content of proline when compared to control. No significant differences were found in the rest of treatments. PA-treated and cold-acclimated (four days at 4 °C) plants accumulated at least 15-fold higher levels of proline. Significant differences four days after PAs application were identified in the leaves of the A plants of both cvs under the effect of Spd and Put.

Increasing cold promoted proline accumulation in all tested variants. Intensified proline accumulation was detected in plants treated with PAs. Under the influence of Put, the proline content increased the most—by 23–38%. Spd treatment resulted in 14–21% and Spm in 4–9% proline content increase compared to control plants.

Under increasing cold conditions, proline levels increased 4.2–6.6-fold in N control plants, 6–10-fold in PA-treated, 1.4-fold in control, and 1.5–2-fold in PA-treated A plants. Despite that, under increasing cold PA applications significantly increased proline levels in N plants; however, they did not reach the proline levels of A plants.

### 2.4. Ethylene Emissions

**Figure 4:**
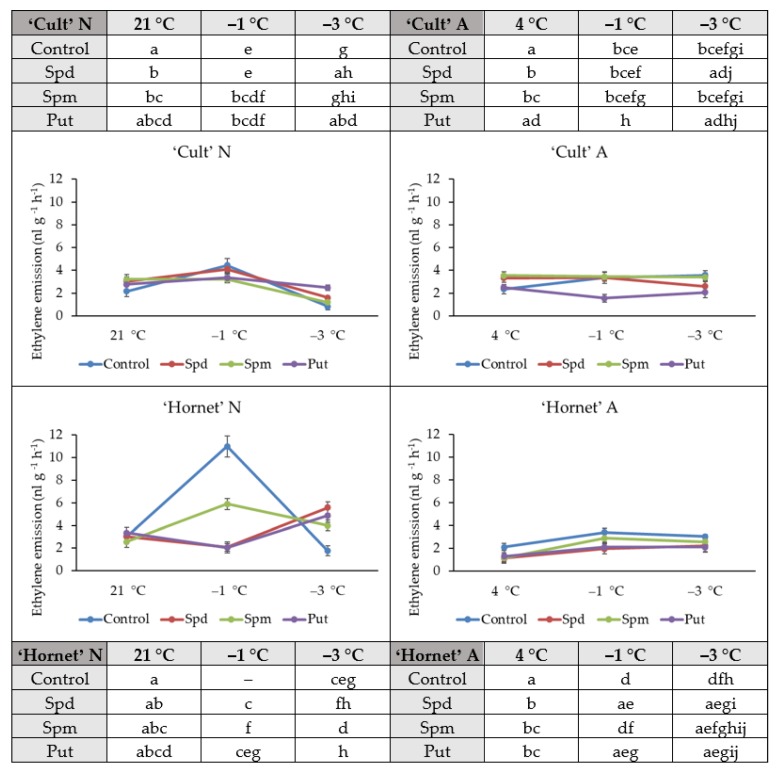
The effect of PA application, cold acclimation (4 °C, 4 days), and increasing cold treatment (from −1 to −3 °C, 2 days) on winter oilseed rape ethylene emission levels. Error bars represent the standard deviation of the mean. Control, Spd, Spm, and Put indicate the type of application. Capital letters N and A indicate non-acclimation and acclimation, respectively. Different lowercase letters indicate statistically significant difference (*p* < 0.05).

After evaluating the influence of exogenous PAs on the ethylene emission of A and N winter oilseed rape leaves, we found that during the increasing cold treatment, ethylene emissions in PA-affected A plants were less intense or close to the control (Figure 4). Significantly higher ethylene emission four days after PAs application was observed in Spd- and Spm-treated both N and A plants of cv. ‘Cult’ and PA-treated A cv. ‘Hornet’ plants as compared to control.

The first day of N plants exposure to cold (−1 °C) showed that the emission of ethylene was lower in PA-treated plants compared to control. Significant differences from control plants were found in Spm- and Put-treated cv. ‘Cult’ (28% and 24%, respectively) and Spd-, Spm- and Put-treated cv. ‘Hornet’ plants (81%, 46% and 81%, respectively). For A plants, significant differences from control plants were found in Put-treated cv. ‘Cult’ (54%) and in Spd- and Put-treated cv. ‘Hornet’ plants (42% and 38%, respectively).

The emission of ethylene of N control plants was lower than that of PA-affected plants after exposure to the frost (−3 °C). The increase percentages of ethylene emissions in cv. ‘Cult’ were 95%, 43%, and 197%, and of cv. ‘Hornet’ were 215%, 126%, and 175%. The ethylene emission of PA-treated A plants was lower than that of the control plants. Significant differences were determined in the Spd- and Put-treated plants of both cultivars. The percentages for cv. ‘Cult’ were 27% and 42%, and for cv. ‘Hornet’, 27% and 31%, respectively.

Overall, PA application resulted in modified winter oilseed rape ethylene emission levels under increasing cold treatment. PAs treatment reduced the ethylene emission of N plants compared to control after the first day of cold (−1 °C) and increased it after the second day of exposure to increasing cold (−3 °C), when compared to control plants. For A plants, application of PAs resulted in lower ethylene emissions than in control plants under increasing cold conditions.

## 3. Discussion

Low temperature negatively affects plant growth, development, and survival [11,16,56]. Exogenous application of physiologically active substances can enhance stress resistance of plants [13,57,58]. In order to improve stress tolerance in crops, different strategies such as foliar application of plant growth regulators, osmoprotectants, and organic and inorganic nutrients have been employed. A number of studies suggest that application of PAs has a positive effect on different abiotic stress tolerance in plants [37,59,60]. However, there is different viewpoint on the relationship between PAs and plant chilling stress [61].

The current study showed that each individual PA (Spd, Spm, and Put) application at a concentration of 1 mM maintained up to 27% higher levels of N winter oilseed rape survival after the increasing frost (from −1 to −3 °C, two days). The highest rates were determined in Put treated N plants compared to controls of both cultivars with different cold resistance, and the strongest effect was on cold sensitive cv. ‘Hornet’ plant recovery. Thus, the use of PAs increased the winter oilseed rape survival rate. So, these results suggest that PAs take part in winter oilseed rape frost tolerance regulation. This is in agreement with other studies showing alleviation of chilling stress (6 °C) by Put in *Anthurium andraeanum* [62] and the increase of cold tolerance in stevia plants by PA supplementation [36]. Despite that, PA application determined a higher survival rate of N oilseed rape under increasing frost conditions, yet the rates of survival of PA-treated plants did not reach the level of A plants. So, the combination of acclimation and PA application resulted in significantly higher rates of survival after the increasing frost. In general, the PAs could protect winter oilseed rape cultivation from the negative impact of increasing cold, especially in the case of a sudden fall of temperature from positive to negative.

Cold tolerance depends on the capability of tissues to accommodate extracellular freezing and survive the accompanying dehydration stress and protect cells from damage by metabolites having osmolyte–cryoprotectant properties [11,35,51,56]. Under cold stress, plants accumulate metabolites, e.g., amino acids such as proline [15]. Proline is an important compatible osmolyte, which maintains turgor, stabilizes cell molecular structure, and acts as a source of energy and nitrogen under various stress conditions [41]. The current study on winter oilseed rape showed that cold treatments promoted proline accumulation in all tested variants. It is suggested that exogenous application of PAs may act as a substrate for proline biosynthesis [1]. In our study, Put-treated N cold-tolerant cv. ‘Cult’ plants accumulated much higher levels of proline when compared to controls (Figure 3). This data supports the findings of Sun and co-authors [62], showing that Put application enhances proline accumulation and alleviates chilling stress (6 °C) in *Anthurium andraeanum*. No significant differences were found in the rest of treatments (N plants). Significant differences were identified in the leaves of Spd- and Put-treated A plants of both cultivars. Under the influence of Put, the proline content increased the most, by 23–38%. Spd treatment resulted in 14–21%, and Spm in 4–9% proline content increase compared to control plants. We confirmed recent studies that exogenous growth regulators can influence the accumulation of proline in plants [10,32]. Furthermore, some reports have indicated that exogenous PAs induced proline accumulation [41,49]. The data of this research showed that foliar application of PAs resulted in intensified proline accumulation in N and A plants under increasing cold (from −1 °C to −3 °C). Under increasing cold conditions, proline levels increased 4.2–6.6-fold in N control plants, 6–10-fold in PA-treated, 1.4-fold in control, and 1.5–2-fold in PA-treated A plants. So, exogenous application of PAs resulted in higher content of proline in both cultivars under increasingly cold conditions. The highest rates of proline were noted in Put-treated plants. Although exposure of N plant to PAs resulted in an increase in proline content during increasing cold stress, it did not reach proline accumulation in untreated A plants. Proline accumulation during acclimation occurs more intensively than under cold stress conditions. Despite that, under cold stress PAs applications significantly increased proline levels in N plants; however, they did not reach the proline levels of A plants. Thus, bearing this in mind, it can be suggested that foliar application of PAs has an acclimating effect on winter oilseed rape plants, and it can be used for protection against frost in their cultivation.

Under stress conditions, the phytohormone ethylene causes growth inhibition, regulates a defense response mostly in full-grown leaves, and a growth response in young leaves [2,25]. It has been observed that concentrations of the plant hormone ethylene under cold/freezing stress conditions depend on plant sensitivity and the growth conditions [63,64,65,66]. Under low temperature, ethylene emissions increase in grapevine, tobacco, tomato, and winter rye [2,11,28], while they decrease in cold-treated *Arabidopsis* [25]. In non-acclimated winter oilseed rape, emissions have been shown to increase, while they are more stable in acclimated plants [16]. What is more, the increased ethylene level under low temperature is thought to be a sign of damage in sensitive plants [24,25,26]. Several studies have shown that the accumulation of ethylene can be influenced by plant resistance increasing substances [10,46]. Additionally, ethylene production in fruits has previously been shown to be inhibited by applications of PAs and having controversial effects on a number of physiological processes [46,47]. There is increasing evidence that PAs, whether applied exogenously, can positively affect plant growth, productivity, and heat as well drought stress tolerance [37], but there is no data on how they can affect ethylene emission in cold and freezing conditions. The current study on winter oilseed rape showed that foliar application of PAs (Spm, Spd, and Put) changed winter oilseed rape ethylene emission levels under cold conditions. The acclimation (four days at 4 °C) and increasing cold (from −1 to −3 °C) treatment modified the levels of ethylene both in PA-non-treated (control) and PA-treated plants (Figure 4). PA treatment reduced the rapid ethylene emission of N plants after the first day of cold (−1 °C) and increased it after the second day of exposure to increasing cold (−3 °C) compared to control plants. Significant differences were found in Spm- and Put-treated cv. ‘Cult’ and Spd-, Spm-, and Put-treated cv. ‘Hornet’ plants as compared to control plants. In this case the ethylene emission remained more stable under increasing cold conditions. This tendency was more evident for A plants; application of PAs resulted in lower than in control plants ethylene emissions under cold stress. Significant differences were found in Put-treated cv. ‘Cult’ and in Spd-, Spm-, and Put-treated cv. ‘Hornet’ plants as compared to controls. Exposure to the frost (−3 °C) increased the ethylene emissions of N and PA-treated plants as compared to control plants. Differently, ethylene emissions of A and PA-treated plants were lower than those of control plants. Significant differences were determined in Spd- and Put-treated plants of both cultivars. So, A and PA-treated plants maintained more stable ethylene emission levels under increasing cold stress. It is important to note that along with this, PAs (Put, Spd, and Spm) increased the survival rate of N winter oilseed rape by 7–27% under increasing frost (from −1 to −3 °C, two days) treatment, and Put showed the strongest effect. We assume that PA treatment delayed the rise in ethylene emissions, thus increasing winter oilseed rape plasticity in response to cold stress.

The cell membrane is considered to be the first possible target of cold. Different studies have indicated that the membrane systems of the cell are the primary sites of freezing injury in plants [20,51,52]. Cold disrupts the membrane and disturbs its activity. In our study, the increasing cold treatment (from −1 to −3 °C, two days) resulted in reduced H^+^-ATPase activity in the A and N winter oilseed rape seedlings; earlier, we found that under the increasing cold to −7 °C, the activity of this enzyme decreased more than two-fold [16]. PAs are known as polycations that interact with cell membrane phospholipids and significantly influence membrane stability, prevent cytolysis, and improve cold resistance [37,55]. We tested PA impact on H^+^-ATPase activity of winter oilseed rape cell membranes and revealed H^+^-ATPase activity to be higher in the PA-treated seedlings after cold exposure than in the control plants. After the exposure to frost, H^+^-ATPase activity in N PA-treated plants was significantly higher when compared to control plants (Figure 2). In the case of A plants, the H^+^-ATPase activity of the control plants was significantly lower than the activity of Spd-treated cv. ‘Cult’ (by 20%) and Put-treated cv. ‘Hornet’ (by 23%) plants.

The obtained results may highlight the mode of action of exogenous PAs in winter oilseed rape. The foliar application of 1 mM PAs (Spd, Spm, and Put) could help plants to invert the adverse effects of cold stress, and might play a key role in providing tolerance in plants through modulating the proline, ethylene content, and H^+^-ATPase activity in winter oilseed rape, thereby increasing survival. The data of the current study on winter oilseed rape revealed that foliar application of PAs may activate a defensive response (act as elicitor to trigger physiological processes), which may compensate the negative impact of cold stress. Generally, PAs can be used as cold stress protective compounds for winter oilseed rape cultivation in temperate climate regions.

## 4. Materials and Methods

### 4.1. Plant Material and Cultivation Conditions

This experiment was carried out under controlled conditions in the laboratory. Open-pollinated cv. ‘Cult’ (medium-early, bred in Sweden) and hybrid ‘Hornet’ (early, bred in Germany) were used in the experiment. Seeds of winter oilseed rape cultivars with different cold tolerance were supplied by the Lithuanian Seeding Association. The seeds were cultivated in plastic cube pots of 10 cm edge length. Each pot contained nine plants. The substrate used in the pots (pH 5.5–6.5) was composed of garden compost and peat moss (1:1 *v/v*) purchased in the store. Substrate was irrigated using a tap water. No fertilizer was used in this study.

**Figure 5:**
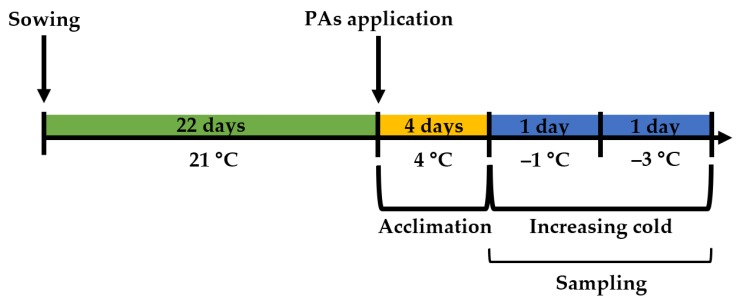
The design of the experiment.

Plants were cultivated for 22 days (Figure 5) until the 3–4 fully expanded leaves stage, BBCH 13–14 [67] in a conditioned growth chamber Climacell (MMM Medcenter Einrichtungen GmbH). The 21 ± 1 °C temperature, photoperiod of 16/8 h day/night, and 60 µmol m^−2^ s^−1^ cool white fluorescent light photon flux at the soil level were set. Subsequent experimental treatments were performed under the same illumination.

### 4.2. Application of PAs

For the preventive effect assessment, pots with seedlings were divided into four groups and labelled as control, Spd, Spm, and Put. Each group consisted of 24 pots with 9 plants in each pot. PA concentration used in this study was chosen based on the other studies [47]. Three groups were foliarly treated with 1 mM water solutions of different types of PAs, namely Spd, Spm, and Put (Sigma). Each group of pots was sprayed with 50 mL of tested PA using a hand sprayer. Control plants were sprayed with the same volume of distilled water. Plants were air-dried for 1 h at a temperature of 21 ± 1 °C and then subjected to temperature treatments.

### 4.3. Temperature Treatments

#### 4.3.1. Cold Acclimation Treatment

Following PAs application, the four-day-long cold acclimation treatment was performed (Figure 5). In order to obtain N and A plants, each of four groups (control, Spd, Spm, and Put) was divided into two subgroups. One subgroup of plants continued growing under previous conditions at 21 ± 1 °C temperature (N plants); another subgroup was transferred to acclimating conditions at a constant temperature of 4 ± 1 °C (A plants).

#### 4.3.2. Increasing Cold Treatment

A two-day-long increasing cold (from −1 to −3 °C) treatment was performed during 27–28th experimental days (Figure 5). Both N and A plants were transferred to Friocell (MMM Medcenter Einrichtungen GmbH) cooling incubators. Then the temperature was gradually lowered by 2 °C h^−1^ until −1 °C was reached. On the second day of the treatment the temperature was reduced by the same rate to −3 °C (Figure 5).

### 4.4. Sampling

The fully expanded leaf blades were collected for assays at the following stages: after cold acclimation and each day of the increasing cold treatment. Freshly harvested samples were used for ethylene emission analysis. Samples collected for H^+^-ATPase activity and proline assays were quickly placed in liquid nitrogen and stored in an ultra-low freezer (Skadi Green line) at −80 °C.

### 4.5. Determination of Plant Survival

The test of survival of plants after cold acclimation and each day of the increasing cold treatment was performed in Climacell plant growing chamber. Pots with cold treated plants were transferred to initial growing conditions (21 °C). Plants in each pot were estimated as dead or living after a 10-day-recovery period [3]. Plant survival was presented as a percentage of recovered plants.

### 4.6. Extraction and Activity Assay of H^+^-ATPase

The differential centrifugation technique was used to extract membrane fractions [21]. Plant material (2.5 g) was homogenized by soft grinding in a previously chilled mortar for 2 min with 1 mL of cold (4 °C) extraction buffer (pH 7.8) containing 1.05 M Tris-HCl (Chempur), 35 mM EDTA (Lachema), 875 mM saccharose (POCH S.A.), and 14 mM DTT (Reanal).

Cotton cloth was used to filter the homogenate. The filtrate was centrifuged for 5 min at 4500× *g* and then for 20 min at 18,000× *g* (centrifuge MPW-351 R). The obtained supernatant was centrifuged for 1 h at 92,200× *g* (centrifuge Thermo Scientific Sorvall WX 100 Ultra, Waltham, MA, USA). The sedimented membrane fraction was collected, resuspended in 1.2 mL of 5 mM Tris–MES (Calbiochem) buffer (pH 7.2), and homogenized in glass potter. The content of H^+^-ATPase (protein) in membrane fraction was evaluated spectrophotometrically at 595 nm wavelength (Analytik Jena SPECORD® 210 PLUS, Jena, Germany) using a Bradford protein assay [68]. Bovine serum albumin (Sigma) was used as a standard. Resuspended membrane fractions with different protein concentrations were calibrated to the appropriate protein concentration by dilution with 1 mM Tris–MES buffer (pH 7.2). The activity of H^+^-ATPase in membrane enriched fractions was evaluated by the release of inorganic phosphate (P_i_), which accumulates due to ATP hydrolysis. The reaction was started by adding 150 µL of membrane suspension (5–20 µg protein) to the medium with the following composition: 3 mM ATP, 3 mM MgSO_4_, 50 mM KCl, and 30 mM Tris–MES (pH 7.2). After 30 min incubation at 37 °C the reaction was stopped by the addition of cooled trichloracetic acid to a final concentration of 3%. P_i_ color reaction with ammonium molybdate and stannous chloride was used. Absorbance was read at 750 nm. The activity of H^+^-ATPase was expressed in µmol P_i_ produced per hour per mg of protein.

### 4.7. Determination of Proline Content

A color reaction of acidified ninhydrin [69] was used to determine the content of proline. The plant material (0.5 g) was grinded in a previously chilled mortar and pestle for 2 min with 10 mL of 3% sulphosalicylic acid (Roth) and extracted at 4 °C for 14 h. Subsequently, extracts were centrifuged at 700× *g* (centrifuge MPW–351 R) for 20 min. Acidified ninhydrin solution was prepared by dissolving ninhydrin (1.25 g) (Roth) in glacial acetic acid (30 mL) (Roth) and 6 M phosphoric acid (20 mL) (SIGMA-ALDRICH Chemie GmbH) by warming and shaking until dissolved. The equal volume of supernatant, acetic acid, and acidified ninhydrin was mixed and heated for 1 h at 100 °C in a heater BLOCKTHERMOSTAT BT 200 (Kleinfeld Labortechnik, Gehrden, Germany). Tubes with hot samples were transferred into an ice bath and chilled for 15 min. Then, the formed chromophore was extracted with toluene (Roth) by vigorous shaking and incubation in the dark for 1 h. The absorbance was read spectrophotometrically at 520 nm using a multi sample quartz cuvette (Hellma) and Rainbow microplate reader (SLT Labinstruments, Rendsburg-Eckernförde, Germany). Toluene was used as a blank. The corresponding content of proline was determined using the standard curve of known L-proline (Roth) concentrations. Calculations were provided using the SLT programme (SLT Labinstruments, Salzburg, Austria). Results were expressed as μmol of proline per g of fresh mass.

### 4.8. Ethylene Emission Assay

A slightly modified method of Child et al. [70] was used to evaluate ethylene emission from leaves. Leaf samples were weighed, placed in 40 mL volume glass vials, and sealed with PTFE/Si septa (Agilent technologies). After 24 hours of incubation at 21 ± 1 °C in the dark, 1 mL of sample gas was manually sampled from each vial using a gas-tight syringe (Agilent Technology, Santa Clara, CA, USA) and injected into a gas chromatograph (Thermo Scientific* FOCUS GC, Waltham, MA, USA) supplied with a stainless-steel column (matrix 80/100 Thermo Scientific* PROPAC R, Waltham, MA, USA) and hydrogen flame ionization detector. The 110, 90, and 150 °C temperatures were set for the injector, column and detector, respectively. The carrier gas was helium (AGA). Calibration was performed using ethylene (Messer) as a standard. Results were expressed as nanoliter of ethylene per g of fresh mass per hour.

### 4.9. Statistical Analysis

This experiment was performed three times. The analyses of the plant material were performed in four replicates. The results were expressed as mean ± standard deviation. The normality of the data was tested using the Shapiro–Wilk test. The data were subjected to the analysis of variance (ANOVA). The multiple comparisons for mean values were performed by the Tukey HSD post hoc test. The differences with *p* values of < 0.05 were considered to be significant.

## 5. Conclusions

Exogenous PA application improved cold resistance, maintained the activity of plasma membrane H^+^-ATPase, increased the content of free proline, and delayed the stimulation of ethylene emission under increasing cold. The results of the current study on winter oilseed rape revealed that foliar application of PAs may activate defensive responses (act as elicitor to trigger physiological processes), which may compensate for the negative impacts of cold stress. Cold tolerance can be enhanced by PA treatment. PAs promoted proline accumulation in the leaves of both A and N winter oilseed rape after exposure to increasing cold stress from −1 to −3 °C. The effect of PAs was more pronounced in A than in N plants. Application of PAs resulted in lower ethylene emissions in plants under cold stress. The combination of acclimation and PA application resulted in significantly higher rates of survival after the increasing frost. The tested substances improved by up to 27% the survival rate of the N rape seedlings. This shows that PAs take part in winter oilseed rape frost tolerance regulation.

## References

[1] Liu J.H., Kitashiba H., Wang J., Ban Y., Moriguchi T. (2007). Polyamines and their ability to provide environmental stress tolerance to plants. Plant Biotechnol..

[2] Zhang Z., Huang R. (2010). Enhanced tolerance to freezing in tobacco and tomato overexpressing transcription factor TERF2/LeERF2 is modulated by ethylene biosynthesis. Plant Mol. Biol..

[3] Fiebelkorn D., Rahman M. (2016). Development of a protocol for frost-tolerance evaluation in rapeseed/canola (*Brassica napus* L.). Crop J..

[4] Kidokoro S., Yoneda K., Takasaki H., Takahashi F., Shinozaki K., Yamaguchi-Shinozaki K. (2017). Different cold-signaling pathways function in the responses to rapid and gradual decreases in temperature. Plant Cell.

[5] McClinchey S.L., Kott L.S. (2008). Production of mutants with high cold tolerance in spring canola (*Brassica napus*). Euphytica.

[6] Paulauskas A., Jodinskienė M., Griciuvienė L., Žukauskienė J., Petraitienė E., Brazauskienė I. (2013). Morphological traits and genetic diversity of differently overwintered oilseed rape (*Brassica napus* L.) cultivars. Zemdirbyste.

[7] Carvalho C.P., Cardoso-Gustavson P., Rodrigues E., Braga M.R., Mercier H., Nievola C.C. (2019). Low temperature acclimation and de-acclimation of the subtropical bromeliad *Nidularium minutum*: Implications of changes in the NO, sugar content and NR activity. Environ. Exp. Bot..

[8] Rapacz M. (2002). Cold-deacclimation of oilseed rape (*Brassica napus* var. *oleifera*) in response to fluctuating temperatures and photoperiod. Ann. Bot..

[9] Ananga A.O., Cebert E., Ochieng J.W., Kumar S., Kambiranda D., Vasanthaiah H., Tsolova V., Senwo Z., Konan K., Anike F.N., Akpan U.G. (2012). Prospects for transgenic and molecular breeding for cold tolerance in canola (*Brassica napus* L.). Oilseeds.

[10] Gavelienė V., Pakalniškytė L., Novickienė L. (2014). Regulation of proline and ethylene levels in rape seedlings for freezing tolerance. Cent. Eur. J. Biol..

[11] Rihan H.Z., Al-Issawi M., Fuller M.P. (2017). Advances in physiological and molecular aspects of plant cold tolerance. J. Plant Interact..

[12] Arora R., Rowland L.J. (2011). Physiological research on winter-hardiness: Deacclimation resistance, reacclimation ability, photoprotection strategies, and a cold acclimation protocol design. HortScience.

[13] Hayat S., Hayat Q., Alyemeni M.N., Wani A.S., Pichtel J., Ahmad A. (2012). Role of proline under changing environments. Plant Signal Behav..

[14] Jakienė E. (2013). The effect of the microelement fertilizers and bio-logical preparation Terra Sorb Foliar on spring rape crop. Žemės ūkio mokslai.

[15] Lehmann S., Funck D., Szabados L., Rentsch D. (2010). Proline metabolism and transport in plant development. Amino Acids.

[16] Jankovska-Bortkevič E., Gavelienė V., Jankauskienė J., Mockevičiūtė R., Koryznienė D., Jurkonienė S. (2019). Response of winter oilseed rape to imitated temperature fluctuations in autumn-winter period. Environ. Exp. Bot..

[17] Trotel-Aziz P., Niogret M.F., Deleu C., Bouchereau A., Aziz A., Larher F.R. (2003). The control of proline consumption by abscisic acid during osmotic stress recovery of canola leaf discs. Physiol. Plant..

[18] Liang X., Zhang L., Natarajan S.K., Becker D.F. (2013). Proline mechanisms of stress survival. Antioxid. Redox Signal..

[19] Kishor P.B.K., Sreenivasulu N. (2014). Is proline accumulation per se correlated with stress tolerance or is proline homeostasis a more critical issue?. Plant Cell Environ..

[20] Martz F., Sutinen M.L., Kiviniemi S., Palta J.P. (2006). Changes in freezing tolerance, plasma membrane H^+^-ATPase activity and fatty acid composition in *Pinus resinosa* needles during cold acclimation and de-acclimation. Tree Physiol..

[21] Darginavičienė J., Pašakinskienė I., Maksimov G., Rognli O.A., Jurkonienė S., Šveikauskas V., Bareikienė N. (2008). Changes in plasmalemma K^+^ Mg^2+^-ATPase dephosphorylating activity and H^+^ transport in relation to freezing tolerance and seasonal growth of *Festuca pratensis* Huds. J. Plant Physiol..

[22] Janicka-Russak M., Shanker A., Venkateswarlu A. (2011). Plant plasma membrane H^+^-ATPase in adaptation of plants to abiotic stresses. Abiotic Stress Response in Plants—Physiological, Biochemical and Genetic Perspective.

[23] Muzi C., Camoni L., Visconti S., Aducci P. (2016). Cold stress affects H^+^-ATPase and phospholipase D activity in *Arabidopsis*. Plant Physiol. Biochem..

[24] Burr K.E., Wallner S.J., Tinus R.W. (1991). Ethylene and ethane evolution during cold acclimation and deacclimation of ponderosa pine. Can. J. For. Res..

[25] Shi Y., Tian S., Hou L., Huang X., Zhang X., Guo H., Yang S. (2012). Ethylene signaling negatively regulates freezing tolerance by repressing expression of CBF and type-A ARR genes in *Arabidopsis*. Plant cell.

[26] Cristescu S.M., Mandon J., Arslanov D., De Pessemier J., Hermans C., Harren F.J. (2013). Current methods for detecting ethylene in plants. Ann. Bot..

[27] Pei H., Wang H., Wang L., Zheng F., Dong C.H., Pandey K.G. (2017). Regulatory function of ethylene in plant responses to drought, cold, and salt stresses. Mechanism of Plant Hormone Signaling Under Stress.

[28] Yu X.M., Griffith M., Wiseman S.B. (2001). Ethylene induces antifreeze activity in winter rye leaves. Plant Physiol..

[29] Ahanger M.A., Akram N.A., Ashraf M., Alyemeni M.N., Wijaya L., Ahmad P. (2017). Plant responses to environmental stresses-from gene to biotechnology. AoB Plants.

[30] Yakhin O.I., Lubyanov A.A., Yakhin I.A., Brown P.H. (2017). Biostimulants in plant science: A global perspective. Front. Plant Sci..

[31] Yang W., Cai T., Ni Y., Li Y., Guo J., Peng D., Yang D., Yin Y., Wang Z. (2013). Effects of exogenous abscisic acid and gibberellic acid on flling process and nitrogen metabolism characteristics in wheat grains. Aust. J. Crop Sci..

[32] Gavelienė V., Pakalniškytė L., Novickienė L., Balčiauskas L. (2018). Effect of biostimulants on cold resistance and productivity formation in winter rapeseed and winter wheat. Ir. J. Agric. Food Res..

[33] Todorova D., Sergiev I., Alexieva V., Karanov E., Smith A., Hall M. (2007). Polyamine content in *Arabidopsis thaliana* (L.) Heynh during recovery after low and high temperature treatments. Plant Growth Regul..

[34] Ebeed H.T., Hassan N.M., Aljarani A.M. (2017). Exogenous applications of polyamines modulate drought responses in wheat through osmolytes accumulation, increasing free polyamine levels and regulations of polyamine biosynthetic genes. Plant Physiol. Biochem..

[35] Fariduddin Q., Varshney P., Yusuf M., Ahmad A. (2012). Polyamines: Potent modulators of plant responses to stress. J. Plant Interact..

[36] Peynevandi K.M., Razavi S.M., Zahri S. (2018). The ameliorating effects of polyamine supplement on physiological and biochemical parameters of *Stevia rebaudiana* Bertoni under cold stress. Plant Prod. Sci..

[37] Chen D., Shao Q., Yin L., Younis A., Zheng B. (2019). Polyamine function in plants: Metabolism, regulation on development, and roles in abiotic stress responses. Front. Plant Sci..

[38] Kusano T., Berberich T., Tateda C., Takahashi Y. (2008). Polyamines: Essential factors for growth and survival. Planta.

[39] Calişkan N., Kadioğlu A., Güler N. (2017). Exogenously applied polyamines ameliorate osmotic stress-induced damages and delay leaf rolling by improving the antioxidant system in maize genotypes. Turk. J. Biol..

[40] Agudelo-Romero P., Bortolloti C., Pais M.S., Al E. (2013). Study of polyamines during grape ripening indicate an important role of polyamine catabolism. Plant Physiol. Biochem..

[41] Pál M., Tajti J., Szalai G., Peeva V., Végh B., Janda T. (2018). Interaction of polyamines, abscisic acid and proline under osmotic stress in the leaves of wheat plants. Sci. Rep..

[42] Sequera-Mutiozabal M.I., Erban A., Kopka J., Al E. (2016). Global metabolic profiling of *Arabidopsis* polyamine oxidase 4 (AtPAO4) loss-of-function mutants exhibiting delayed dark-induced senescence. Front. Plant Sci..

[43] Zhen Y., Li S., Su X. (2000). Polyamine changes and chilling injury in cold-stored Loquat fruits. Acta Bot. Sin..

[44] Roy M., Wu R. (2001). Arginine decarboxylase transgene expression and analysis of environmental stress tolerance in transgenic rice. Plant Sci..

[45] Wang Y., Lu W., Zhang Z. (2003). ABA and putrescine treatment alleviate the chilling damage of banana fruit. J. Plant Physiol. Mol. Biol..

[46] Bal E. (2013). Effects of exogenous polyamine and ultrasound treatment to improve peach storability. Chil. J. Agric. Res..

[47] Yousefi F., Jabbarzadeh Z., Amiri J., Rasouli-Sadaghiani M.H. (2019). Response of roses *(Rosa hybrida* L. ‘Herbert Stevens’) to foliar application of polyamines on root development, flowering, photosynthetic pigments, antioxidant enzymes activity and NPK. Sci. Rep..

[48] Blum A. (2017). Osmotic adjustment is a prime drought stress adaptive engine in support of plant production: Osmotic adjustment and plant production. Plant Cell Environ..

[49] El Sabagh A., Hossain A., Barutcular C., Gormus O., Ahmad Z., Hussain S., Islam M.S., Alharby H., Bamagoos A., Kumar N. (2019). Effects of drought stress on the quality of major oilseed crops: Implications and possible mitigation strategies. Appl. Ecol. Environ. Res..

[50] Pearce S., Zhu J., Boldizsár Á., Vágújfalvi A., Burke A., Garland-Campbell K., Galiba G., Dubcovsky J. (2013). Large deletions in the CBF gene cluster at the Fr-B2 locus are associated with reduced frost tolerance in wheat. Theor. Appl. Genet..

[51] Sanghera G.S., Wani S.H., Hussain W., Singh N.B. (2011). Engineering cold stress tolerance in crop plants. Curr. Genom..

[52] Guo X., Liu D., Chong K. (2018). Cold signaling in plants: Insights into mechanisms and regulation. J. Integr. Plant Biol..

[53] Barrero-Sicilia C., Silvestre S., Haslam R.P., Michaelson L.V. (2017). Lipid remodelling: Unravelling the response to cold stress in *Arabidopsis* and its extremophile relative *Eutrema salsugineum*. Plant Sci..

[54] Sampedro J.G., Muñoz-Clares R.A., Uribe S. (2002). Trehalose-mediated inhibition of the plasma membrane H^+^-ATPase from *Kluyveromyces lactis*: Dependence on viscosity and temperature. J. Bacteriol..

[55] Li Y., He J. (2012). Advance in metabolism and response to stress of polyamines in plant. Acta Agric Boreali Sin..

[56] Fowler D.B., Byrns B.M., Greer K.J. (2014). Overwinter low-temperature responses of cereals: Analyses and simulation. Crop Sci..

[57] Gill S.S., Tuteja N. (2010). Reactive oxygen species and antioxidant machinery in abiotic stress tolerance in crop plants. Plant Physiol. Biochem..

[58] Bhandari S.R., Kim Y.H., Lee J.G. (2018). Detection of temperature stress using chlorophyll fluorescence parameters and stress-related chlorophyll and proline content in paprika (*Capsicum annuum* L.) seedlings. Hortic. Sci. Technol..

[59] Hummel I., Couée I., El Amrani A., Martin-Tanguy J., Hennion F. (2002). Involvement of polyamines in root development at low temperature in the subantarctic cruciferous species *Pringlea antiscorbutica*. J. Exp. Bot..

[60] Toupchi Z., Lisar S.Y., Ghassemi-Golezani K., Motafakkerazad R. (2018). Physiological Responses of safflower to exogenous putrescine under water deficit. J. Stress Physiol. Biochem..

[61] Wu M., Yuan L. (2008). Research progress in the relationship between polyamine and plant resistance. J. Anhui Agric. Sci..

[62] Sun X., Wang Y., Tan J., Al E. (2018). Effects of exogenous putrescine and D-Arg on physiological and biochemical indices of anthurium under chilling stress. Jiangsu J. Agric. Sci..

[63] Zhao D., Shen L., Fan B., Yu M., Zheng Y., Lv S., Sheng J. (2009). Ethylene and cold participate in the regulation of LeCBF1 gene expression in postharvest tomato fruits. FEBS Lett..

[64] Catala R., Lopez-Cobollo R., Mar Castellano M., Angosto T., Alonso J.M., Ecker J.R., Salinas J. (2014). The *Arabidopsis* 14-3-3 protein RARE COLD INDUCIBLE 1A links low-temperature response and ethylene biosynthesis to regulate freezing tolerance and cold acclimation. Plant Cell.

[65] Zhao M., Liu W., Xia X., Wang T., Zhang W.H. (2014). Cold acclimation-induced freezing tolerance of *Medicago truncatula* seedlings is negatively regulated by ethylene. Physiol. Plant..

[66] Kazan K. (2015). Diverse roles of jasmonates and ethylene in abiotic stress tolerance. Trends Plant Sci..

[67] Weber E., Bleiholder H., Lancashire P.D., Langelüddecke R., Stauss R., Van der Boom T., Witzen-Berger A., Meier U. (2018). Growth stages of mono- and dicotyledonous plants. BBCH Monograph.

[68] Bradford M.M. (1976). A rapid method for the quantification of microgram quantities of proteins utilising the principle of protein-dye binding. Anal. Biochem..

[69] Bates L.S., Waldren R.P., Teare I.D. (1973). Rapid determination of free proline for water-stress studies. Plant Soil.

[70] Child R.D., Chauvaux N., John K., Van Onckelen H.A., Ulvskov P. (1998). Ethylene biosynthesis in oilseed rape pods in relation to pod shatter. J. Exp. Bot..

